# The State-of-the-art of Research into Human Multitasking: An Editorial

**DOI:** 10.5334/joc.185

**Published:** 2021-09-09

**Authors:** Leif Johannsen, Hermann Müller, Andrea Kiesel, Iring Koch

**Affiliations:** 1 Institute of Psychology, RWTH Aachen University, DE; 2 Department of Sport Science, University of Gießen, DE; 3 Department of Psychology, University of Freiburg, DE

**Keywords:** Action, Cognitive Control, Attention

In everyday life, we use the term multitasking to describe situations in which we perform two or more tasks simultaneously. Yet, for a scientific definition, the exact meaning of the terms “task” (Kiesel et al., 2010) and “simultaneous performance” is unclear. Thus, we recently suggested a broad definition of multitasking, as a condition in which cognitive processes involved in multiple tasks overlap in time (Koch et al., 2018). Such a definition of multitasking entails a wide range of research paradigms in the domains of human cognitive psychology and movement sciences. Cognitive psychology has mainly focused on structural and functional limitations of cognitive processes when facing multitasking requirements. Structural limitations assume strict serial processing for at least one processing stage, while functional limitations assume flexible, parallel processing only limited by the number of available resources. Human movement science, on the other hand, emphasizes the plasticity of cognition and training possibilities. As both approaches have provided ample empirical evidence for their views but have predominantly worked in isolation, this example clearly illustrates the need for a more integrative approach to multitasking. A challenge for the contemporary research on multitasking is to bring together the issues of structure, flexibility, and plasticity in human multitasking, offering a new integrative theoretical framework that accounts for this fundamental aspect of human behaviour.

The 16 articles, 2 review articles and 14 research papers, in this special collection cover current research into a diverse range of thematic issues such as cognitive control in multitasking situations, the benefit of learnt sequence knowledge in multitasking, process interference during cognitive-motor dual-tasking, cognitive-postural multitasking training and cognitive task switching. A state-of-the-art theoretical foundation is laid by the two review articles that focus on current efforts to generate process models of human multitasking. In the review by Hommel (2020), the author criticizes the previous research into human information processing bottlenecks with respect to an alleged lack of advancement in scientific theory. He calls for a marriage between theories assuming information processing stages with resource theories. Consequently, Hommel (2020) applies the Theory of Event Coding to dual-task performance in order to bridge the gap between the empirical generalization of processing bottlenecks and the theoretical understanding of the underlying mechanisms. The review by Vandierendonck (2021) provides a conceptual overview about a computational model of working memory processes with distributed executive control, which he then applies to the prediction of costs in task switching, attention switching, and dual-task coordination, as well as in contexts that combine all three aspects. In this respect, these two theoretical frameworks provide the background to the remaining 14 empirical studies introducing different important concepts of multitasking like processing bottlenecks, central resources and working memory.

In everyday life, multitasking often occurs in situations, in which a cognitive task is performed simultaneously with a movement task, such as talking and walking (e.g. Beurskens & Bock, 2012). If the processing difficulties are high and people can decide freely then they will certainly prefer to avoid the requirement to coordinate multiple tasks at the same time, as observed in older adults (e.g. Cohen & Verghese, 2019). If multitasking cannot be avoided, however, then a context-specific, flexible allocation of processing capacity might become necessary to control interference between a cognitive and a movement task. In continuous cyclical movement tasks, it may be therefore a good strategy to allow cognitive processing when the cognitive demands of the movement tasks are low. Assuming that cognitive demands of walking increase in phases depending on the gait cycle, Langhanns and Mueller (2020) investigated cognitive-motor interference during continuous walking with periodic processing demands of an intermittent working memory task. The cognitive task was linked to specific events in the gait cycle. The authors found evidence of prescheduling of processing resources during the gait cycle as expected. As a result of this phasic regulation, however, dual-task processing demands lead to increased interference when the cognitive stimulus had to be processed earlier than expected.

Effortless cognitive-motor multitasking is an ability that suffers as we are getting older, which could have serious health implications due to an increased risk of fall-related injury in older adults. In the context of cognitive-postural multitasking, the article by Brahms et al. (2021) evaluated the effect of a modality-specific, multitasking training intervention in older adults, which utilized the performance benefit of modality compatibility mappings. A six-weeks programme with difficulty progression on both the cognitive and postural tasks compared gains in balance performance between a group that practised with modality compatible task mappings and a group with modality incompatible modality mappings. Surprisingly, improvements in the cognitive task performance and in dual-tasks costs was seen in both groups following a passive control period leading the intervention period but no further gains were observed following the intervention in neither of the two groups. The study highlights the potential challenges and disappointments of intervention research but also the critical importance of publishing negative results in this field of research.

While practicing performance in a multitasking situation is one possible way for reducing dual-tasking costs, predicting the phasic demands of a primary task may be another way to reduce the costs of multitasking. Therefore, sequential learning is a paradigm that demonstrated the plasticity of processes engaged in multitasking. Pfeifer et al. (2021) tested whether the retrieval of a sequence learnt when performing an elbow-centred movement task would be affected by dual-tasking demands imposed by a simple reaction time task. However, a performance-reducing influence of the secondary task was not found during sequence retrieval, which the authors explained with the availability of an abstract representation of the sequence, which makes the primary task invulnerable to dual-tasking interference. Röttger et al. (2021) investigated the disruption of learning in a visual-manual serial reaction time task by interference with a secondary auditory-vocal task. By creating probabilistic pairings between stimulus features in the serial-reaction time task and in the secondary task, the authors demonstrated that across-task contingencies facilitate learning in the primary task despite any imposed dual-task demands. Bröker et al. (2020) assessed motor learning in a continuous tracking task while being engaged in a secondary auditory reaction task. Regularities in the tracking trajectory were acquired in the course of several practice sessions, so that performance benefited from the predictability of the trajectories. Dual-tasking demands were not found to infer with the motor learning. Ewolds et al. (2021) used a similar paradigm to Bröker et al. (2020) to assess transfer of sequential predictability between a visual-manual tracking task and an auditory task. They aimed to assess whether sequential predictability would affect the costs of dual-tasking and observed only task-specific predictability effects such as that dual-task costs in the tracking task were reduced by predictability in the secondary tasks.

Conflict between contradicting response tendencies due to ambiguous targets is a specific multitasking context, in which cognitive control processes are involved in the resolution of any indiced response conflicts. To better understand cognitive control, Dignath et al. (2021) investigated the control mechanisms behind the so called congruence sequence effect, which expresses a reduced congruency effect following an exposure to an incongruent trial. Assuming that either temporal selection as well as temporal order or perceptual features play an important role in cognitive control, the temporal order of primes and targets were counterbalanced and contrasted. The authors observed a greater congruence sequence effect when the same prime-target order was repeated in subsequent trials which suggests that temporal selection is important for cognitive control.

Certain response tendencies are also triggered by the modality of any target stimuli, so that visual stimuli facilitate a manual response, while auditory stimuli favour a vocal response. Koob et al. (2020) investigated compatibility-based backward crosstalk effects based on perceptual overlap between two tasks. Their results contradict the assumption that backward crosstalk effects are caused by perceptual overlap. Instead, the authors suggest a model, which proposes that central capacity limitations underlie this phenomenon.

Task switching resembles a dual-task situation insofar as it leads to the involuntary activation of competing task sets in working memory. Therefore, it is the “cream of the crop” discipline that requires effective cognitive control to minimize the costs of task interference. Stephan et al. (2021) examined the costs of task switching with stimulus-response modality-incompatible tasks (auditory-manual and visual-vocal) depending on the generality of modality compatibility and the frequency of task switches. The frequency of switches did not influence the switching costs compared to modality-compatible tasks but modality-compatibility effects generalized from manual to pedal responses. Based on this latter finding, the authors argue that competing modality mappings based on ‘‘backward’’ linkages between anticipated response effects and stimuli lead to modality compatibility-dependent switching costs. By focusing on n-2 task-repetition costs and in order to translate traditional experimental paradigms into contexts and settings that more closely resemble everyday life situations, Schuch et al. (2020) investigated the importance of inhibitory processes in task switching in the situational context of complex visual scenes depicting the perspective of a car driver. They observed n-2 task repetition costs only when target stimuli were not surrounded by a complex visual scene as in traditional experiments. They argue that the complexity of the visual background leads to an attenuation of n-2 task repetition costs. Aufschnaiter et al. (2021) demonstrate that task switches and repetitions can be prepared if they become predictable by the duration of pre-target intervals. The authors propose a two-stage preparation model according to which task switches and repetitions are prepared in an unspecific manner, such as by previous task set inhibition, based on expectancies derived from the duration of intertask transitions. Elchlepp et al. (2021) investigated switching between task sets using a neurophysiological approach by recording event-related cortical potentials. They used visual compound letter-on-face stimuli and cue instructed participants to evaluate either the letters or faces. The difference between classification of neutral or emotional faces in terms of event-related potentials was not influenced by task switches or repetitions, so that the authors conclude that judging a facial expression is an involuntary process that does not share the properties of cognitive task sets.

Switching between two different language sets in a conversation is an impressive multitasking skill, especially when it appears immediate without any noticeable interruptions, as everybody who as acquired a second language can testify. While true multi-linguals may not demonstrate any difficulties in switching languages, the “non-native” speaker may perceive it as quite challenging to prevent intrusions of their mother tongue when speaking the secondary language. Segal et al. (2021) suggested bilingual language use might rely on processes involved in non-linguistic multitasking. In order to develop a procedure for testing their hypothesis, the authors studied the reliability and consistency of both switching and mixing costs in the context of bilingualism asking whether language-based tasks would be subject to similar individual differences as observed in non-linguistic colour-shape tasks. Comparable reliability and consistency properties of switching and mixing costs were confirmed between the linguistic and non-linguistic tasks so that correlations between both task domains could be tested.

In everyday life, we need to organize several activities as efficiently as possible, for example coordinating work and family duties while working from home during a global pandemic. Often, we need to consider what has to be finished urgently, decide which task can be postponed, keeping in mind the temporal effort required perform a certain task successfully. Monno et al. (2021) asked participants to self-organize switching between tasks with the constraint, however, that repeating a task would gradually increase the waiting time before the target stimulus for each repetition. In contrast, switching the task would make the target available immediately. The authors manipulated two features of a trial, the switch costs by the intertrial interval and the increment in waiting time for consecutive repetitions, to assess the balance between these two factors that participants would voluntarily adopt. They found that participants would opt for an “optimal” balance with medium switch costs and small waiting time increments.

Overall, this special collection provides the reader with a diverse cross-section of the most prominent and important approaches in multitasking research considering cognitive and motor task requirements. It also shows us where we are at the moment in our research endeavour and where we might go next.
